# Evaluation of Two Primer Sets for Amplification of Comammox *Nitrospira amoA* Genes in Wetland Soils

**DOI:** 10.3389/fmicb.2020.560942

**Published:** 2020-09-30

**Authors:** Chenshuo Lin, Hang Xu, Wei Qin, Shaoyi Xu, Xiufeng Tang, Lu Kuang, Xinxin Wang, Bin Jiang, Junhui Chen, Jun Shan, Jonathan Adams, Hua Qin, Baozhan Wang

**Affiliations:** ^1^State Key Laboratory of Subtropical Silviculture, Zhejiang A&F University, Hangzhou, China; ^2^Key Laboratory of Integrated Regulation and Resource Development on Shallow Lake of Ministry of Education, College of Environment, Hohai University, Nanjing, China; ^3^Department of Microbiology and Plant Biology, University of Oklahoma, Norman, OK, United States; ^4^Key Laboratory of Environment Remediation and Ecological Health, Ministry of Education, College of Environmental Resource Sciences, Zhejiang University, Hangzhou, China; ^5^State Key Laboratory of Soil and Sustainable Agriculture, Institute of Soil Science, Chinese Academy of Sciences, Nanjing, China; ^6^Agricultural Genomics Institute at Shenzhen, Chinese Academy of Agricultural Sciences, Shenzhen, China; ^7^School of Geography and Ocean Science, Nanjing University, Nanjing, China; ^8^Key Laboratory of Microbiological Engineering of Agricultural Environment, Ministry of Agriculture, College of Life Sciences, Nanjing Agricultural University, Nanjing, China

**Keywords:** comammox, *amoA*, Ntsp-amoA 162F/359R, comaA/B-244f/659r, PCR

## Abstract

After the discovery of complete ammonia-oxidizing (comammox) *Nitrospira*, detection and assessments of the contribution of comammox *Nitrospira* communities to nitrogen cycling are in great demand. PCR-based approach, a common method for the detection of comammox, depends strongly on accurate amplification of the *amoA* genes from the original DNA samples using appropriate primers. In this study, we reported an evaluation of the performance of two commonly used primer sets, Ntsp-amoA 162F/359R and comaA/B-244f/659r, for amplifying the comammox *amoA* genes from three representative wetland soils in China [Sangsang (SS), Sanjiang (SJ), and Xianghai (XH)]. Our results demonstrated the two primer sets could both successfully amplify the clades with high relative abundances (RA), and further revealed a broadly similar diversity and community composition of dominant comammox operational taxonomic units (OTUs) (RA ≥ 1%) in each of the three wetland soils. However, the clades with low RA, such as the clade A (1.26%) in SJ and the clade B (11.54%) in XH that were recovered by metagenomics analysis, failed to be amplified using comaA/B-244f/659r, but were successfully amplified and sequenced using Ntsp-amoA 162F/359R. It indicated that, compared to comaA/B-244f/659r, Ntsp-amoA 162F/359R was more sensitive to the clades with low RA. However, it is worth noting that Ntsp-amoA 162F/359R would overestimate the RA of some rare clades. For example, the RAs of clade B in XH were overestimated by 32-fold. Furthermore, high levels of non-target amplification were detected via gel electrophoresis using both primer sets, especially for comammox Clade B *amoA* genes, implying that we should treat qPCR results based on these primers with caution. Taken together, our study comprehensively compared the performance of the two primer sets on the sensitivity and specificity of amplifying comammox *amoA* genes in three wetland soils, pointing out the necessity of further development of new primers for the efficient and accurate detection of comammox in various environments.

## Introduction

Nitrification, the aerobic oxidation of ammonia via nitrite to nitrate, is an essential part of the global nitrogen (N) cycling process (Galloway et al., 2008). It was originally considered to be a two-step process catalyzed by two distinct functional groups of microorganisms, the ammonia-oxidizing microbes (AOM) including ammonia-oxidizing bacteria (AOB) and ammonia-oxidizing archaea (AOA) (Könneke et al., 2005), and nitrite-oxidizing bacteria (NOB). This perception was overturned in 2015 with the surprising discovery of complete ammonia oxidizers (comammox) (Daims et al., 2015; van Kessel et al., 2015), which encode all the necessary enzymes involved in ammonia oxidation and nitrite oxidation in their genomes and perform complete nitrification activity in a single organism (Santoro, 2016).

Comammox of the genus *Nitrospira* are widely distributed in both anthropogenic and natural habitats, including recirculating aquaculture systems (Bartelme et al., 2017), drinking water distribution systems (Pinto et al., 2016; Wang Y. et al., 2017), groundwater-fed rapid sand filters (Palomo et al., 2016; Bartelme et al., 2017; Fowler et al., 2018), pipe surface (Daims et al., 2015), wastewater treatment plants (Chao et al., 2016; Camejo et al., 2017; Fan et al., 2017; Annavajhala et al., 2018; Wu et al., 2019), estuarine waters, leaf surface and agricultural soils (Orellana et al., 2018), terrestrial subsurface (Poghosyan et al., 2019), forest soils (Pjevac et al., 2017), and freshwater environments (wetlands, river waters, lake waters, aquifers and lake sediments) (Yu et al., 2018; Jiang et al., 2019). These studies have highlighted considerable phylogenetic diversity among comammox *Nitrospira*. So far, the existing comammox *Nitrospira* sequences can be assigned to two clades, designated as clades A and B (Daims et al., 2015). Clade A is further divided into clades A.1 and A.2 (Xia et al., 2018). However, current knowledge about the ecological distribution of phylotypes and their potential contribution to nitrification is still limited.

Molecular surveys of the environmental distribution of comammox *Nitrospira* are primarily based on PCR amplification of the *amoA* gene that codes for the alpha subunit of ammonia monooxygenase (AMO) (Purkhold et al., 2000; Pester et al., 2011), the key enzyme of aerobic ammonia oxidation. However, the AMO of comammox *Nitrospira* represents a novel type of AMO that is distinct from its counterparts among the known AOB and AOA (Pjevac et al., 2017). Thus, the established primer sets that target the *amoA* genes of AOB or AOA cannot be used for the detection of comammox *amoA* genes in environments. Consequently, several primer sets specific for comammox *amoA* genes have been developed, including separate primer collections for clade A and clade B of comammox (Pjevac et al., 2017), a primer pair of high coverage simultaneously targeting all comammox within *Nitrospira* (Fowler et al., 2018) and a two-step PCR protocol primer giving broad coverage of copper containing membrane monooxygenase genes (Wang J.G. et al., 2017). Other newly developed primers only detect a certain fraction of the comammox *amoA* genes within the clade A (Bartelme et al., 2017; Wang et al., 2018; Xia et al., 2018; Beach and Noguera, 2019; Zheng et al., 2019). Among them, the primer sets of Ntsp-amoA 162F/359R (Fowler et al., 2018) and comaA/B-244f/659r (Pjevac et al., 2017) are the two most widely used primers for studying the environmental distribution of comammox *amoA* genes (Wang Y. et al., 2017; Fowler et al., 2018; Shi et al., 2018; Roots et al., 2019; Spasov et al., 2019; Wang et al., 2019). An appropriate primer set is crucial for achieving an unbiased representation and efficient assessment of comammox community composition in various environments. However, it is not yet clear if there are major differences between these two primer sets in terms of PCR amplification efficiency and specificity.

In this study, we investigated two widely used primer sets, comaA/B-244f/659r (Pjevac et al., 2017) and Ntsp-amoA 162F/359R (Fowler et al., 2018) targeting the comammox *amoA* genes. We used high-throughput amplicon sequencing and metagenomics sequencing on the Illumina platforms to characterize the composition and structure of comammox community in three typical wetland soils from the Tibetan Plateau and Northeast Plain in China. The amplification efficiency and specificity of the two primer sets was evaluated based on the comparative analysis of the phylogenetic distribution of comammox *amoA* amplicons and *amoA* fragments from directly sequenced metagenomes. The aim of this study was to assess the degree of caution that should be exercised when interpreting the comammox communities results obtained from the two PCR primers (Ntsp-amoA 162F/359R and comaA/B-244f/659r).

## Materials and Methods

### Sample Collection and DNA Extraction

Soil samples were collected in August 2016 from Sanjiang (SJ) wetland (47°35′N, 133°38′E) and Xianghai (XH) wetland (44°35′N, 122°33′E) in the Northeast Plain and from Sangsang (SS) wetland (29°22′N, 86°31′E) in the Qinghai-Tibet Plateau. Bulk soil at a depth of 0–10 cm were collected in triplicate from each site. Soil samples were immediately transported on ice to the laboratory and stored at −20°C. The soils were freeze-dried and passed through a 2-mm sieve and then homogenized for DNA extraction. Total DNA was extracted using a FastDNA^®^ spin kit (MP Biomedicals, Cleveland, OH, United States) according to the manufacturer’s instructions. Quantity and concentration of soil DNA were measured by a NanoDrop ND-1000 UV–vis spectrophotometer (NanoDrop Technologies, Wilmington, DE, United States).

### PCR Amplification of Comammox *amoA* Genes

**FIGURE 1:**
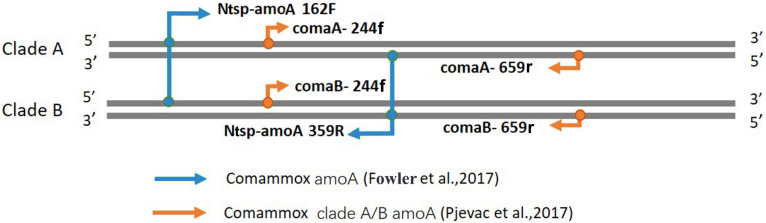
Visual representation of primer-binding sites with comammox clade A *amoA* genes and comammox clade B *amoA* genes. The primer set comaA/B -244f/359r, labeled with orange arrows, are located between positions 244–261 and 643–659 in comammox *amoA* genes. The Amplification with primers Ntsp-amoA 162F/359R (blue arrows) occurs at binding regions between positions between 162–182 and 339–359.

Amplification of comammox *amoA* genes was performed using the primer mixtures comaA/B-244f/659r and the primer set of Ntsp-amoA 162F/359R (Figure 1 and Table 1) on a T100 thermal cycler (Bio-Rad, United States). A 10-bp-long unique barcode was added to the forward primers to distinguish the samples. PCRs of total comammox *amoA* genes (Fowler et al., 2018) were performed in triplicate at a volume of 50 μl: 25 μl of 2 × Green Taq Mix (Vazyme, Nanjing, China), 2 μl of each primer (10 μM) and 10 ng of template DNA. Thermal cycling was carried out with an initial denaturation step at 95°C for 3 min followed by 30 cycles of initial denaturation at 95°C for 30 s, annealing at 48°C for 20 s, extension at 72°C for 30 s and a final elongation step at 72°C for 5 min.

**TABLE 1:** Properties of the comammox amoA-targeted primer sets used for PCR in this study.

**Target gene**	**Primer name**	**Forward primer (5′-3′)^a^**	**Degeneracy**	**Position^b^**	**Approximate product size (bp)**	**References**
Comammox clade A *amoA*	comaA-244f_a comaA-244f_b comaA-244f_c comaA-244f_d comaA-244f_e comaA-244f_f comaA-659r_a comaA-659r_b comaA-659r_c comaA-659r_d comaA-659r_d comaA-659r_f	TACAACTGGGTGAACTA TATAACTGGGTGAACTA TACAATTGGGTGAACTA TACAACTGGGTCAACTA TACAACTGGGTCAATTA TATAACTGGGTCAATTA AGATCATGGTGCTATG AAATCATGGTGCTATG AGATCATGGTGCTGTG AAATCATGGTGCTGTG AGATCATCGTGCTGTG AAATCATCGTGCTGTG	1 1 1 1 1 1 1 1 1 1 1 1	244–261	415	Pjevac et al., 2017
				643–659		
Comammox clade B *amoA*	comaB-244f_a comaB-244f_b comaB-244f_c comaB-244f_d comaB-244f_e comaB-244f_f comaB-659r_a comaB-659r_b comaB-659r_c comaB-659r_d comaB-659r_d comaB-659r_f	TA**Y**TTCTGGACGTTCTA TA**Y**TTCTGGACATTCTA TACTTCTGGACTTTCTA TA**Y**TTCTGGACGTTTTA TA**Y**TTCTGGACATTTTA TACTTCTGGACCTTCTA A**R**ATCCAGACGGTGTG A**R**ATCCAAACGGTGTG A**R**ATCCAGACAGTGTG A**R**ATCCAAACAGTGTG AGATCCAGACTGTGTG AGATCCAAACAGTGTG	2 2 1 2 2 1 2 2 2 2 1 1	244–261	415	
				643–659		
Total Comammox *amoA*	Ntsp-amoA-162F Ntsp-amoA-359R	GGATTTCTGG**N**T**S**GATTGGA **W**AGTT**N**GACCACCA**S**TACCA	8 16	162–182 339–359	198	Fowler et al., 2018

*^*a*^Degenerate (in bold): R, A/G; Y, C/T; S, C/G; W, A/T; N, A/T/C/G. ^*b*^Position is based on the *Nitrospira* sp. SG-bin1 amoA (GenBank, WP_011635097).*

Amplifications of clade A and clade B comammox *amoA* genes (Pjevac et al., 2017) were performed with individual equimolar primer mixtures according to Pjevac et al. (2017) (Figure 1 and Table 1). The PCR reactions were performed as described above with the following thermocycling protocol: 95°C for 3 min, 30 cycles of 95°C for 15 s, 52°C for 20 s, and 72°C for 30 s, and a final elongation step 72°C for 5 min. After running on the 2% agarose gels, all PCR products of the potentially correct length were purified with an AxyPrep gel extraction kit (AxyGen, Hangzhou, China). NanoDrop ND-1000 spectrophotometer was used to quantify the purified amplicons.

### High Throughput Sequencing and Analysis

The purified amplicons using primer set Ntsp-amoA 162F/359R were sequenced on an Illumina HiSeq platform (2 × 150 bp, Illumina, San Diego, CA, United States). The amplicons of the clade A and clade B comammox *amoA* genes using comaA/B-244f/659r were subjected to paired-end sequencing (2 × 300 bp) on an Illumina MiSeq platform (Illumina, San Diego, CA, United States) by Shanghai Majorbio Bio-Pharm Technology Co., Ltd. (Shanghai, China).

Illumina data were checked for quality using FastQC^1^ and trimmed in Quantitative Insight into Microbial Ecology (QIIME)^2^ (Zhou et al., 2016), where sequences were filtered based on their Phred quality scores (*Q* = 20). Sequences amplified by comaA/B-244f/659r and Ntsp-amoA 162F/359R were removed if they were shorter than 300 and 150 bp, respectively. Chimeras were identified and eliminated based on both *de novo* and comammox *amoA* reference database^3^ (Edgar, 2010; Zhou et al., 2016). The remaining sequences were clustered into operational taxonomic units (OTUs) with UCLUST based on a 95% identity threshold. The representative sequence of each OTU was determined by blasting against NCBI-nr database, and the sequences with blast hits of comammox *amoA* genes or *pmoA* genes (potential comammox *amoA* gene but wrongly annotated as *pmoA* gene before 2015, since the two primer sets could not generate real AOA or AOB *amoA* genes or *pmoA* genes) were kept and exclusively determined as comammox *amoA* genes in the following phylogenetic analysis. To take advantage of the full potential of the data, Illumina data was also analyzed in QIIME 2 which provides quality control, merging of paired ends, and error correction with DADA2 (Callahan et al., 2016) to remove or correct sequencing reads with errors or chimeras and outputs the abundance of error-corrected amplicon sequence variants (ASVs).

### Phylogenetic Analysis

The representative sequence of each OTU that clustered with the comammox *amoA* genes along with an outgroup of bacterial *amoA* were aligned using MEGA 6.0 (Tamura et al., 2013). Aligned sequences were employed to construct the maximum likelihood by IQ-TREE^4^ with 1,000 bootstrap iterations and neighbor-joining tree by MEGA 6.0 (Tamura et al., 2013).

### Metagenomic Sequencing and Analysis

The genomic DNA was extracted from each wetland soil as described above and sequenced on the Illumina HiSeq 4000 platform with a paired-end strategy (2 × 150 bp with an insert size of 350 bp) at Shanghai Majorbio Bio-Pharm Technology Co., Ltd (Shanghai, China). All raw reads were quality trimmed and filtered using Sickle^5^ with default parameters. A total of ∼40.0 Gbp of clean reads was obtained for each sample. Due to the very low coverage of each comammox genome in the metagenomic dataset, it was impossible to uncover with accuracy the community structures of comammox based on *amoA* genes assembled from the metagenome data [only 2–5 comammox *amoA* genes ≥338 bp were obtained by metagenomic assembly using metaSPAdes v3.13.0 (Nurk et al., 2017) from each sample]. Therefore, we used a hybrid annotation strategy for the short-read screening as described in Liu et al. (2020) to determine the RAs of clade A and clade B in each soil sample. Briefly, clean metagenomic reads were analyzed by BLASTx against the comammox *amoA* gene database from Liu et al. (2020) with E value ≤ 1 × 10^–5^. A read was annotated as a corresponding comammox *amoA*-like sequence only if its BLAST hit had ≥80% amino acid read identity for ≥25 amino acids.

### Statistics Analysis

The Shannon index, OTU richness (with RA ≥ 0.0%, ≥0.01%, ≥0.1%, ≥0.5%, ≥1% of the total comammox *amoA* sequences) and phylogenetic diversity (PD) were calculated using the QIIME package based on OTU thresholds of 95%. To compare diversity indices of wetland samples with different primer sets, one-way analysis of variance (ANOVA) was applied using Statistical Package of Social Sciences (SPSS, version 20.0). *P* values less than 0.05 were considered as statistically significant for all statistical test. To explore the potential similarity of sequences obtained by two primer sets, Compute Pairwise Distance with MEGA 6.0 (Tamura et al., 2013) was applied to calculated similarity of the overlapped areas from OTU representative sequences captured by different primer sets.

### NCBI Sequence Accession Numbers

Amplicon data are deposited in NCBI Sequence Read Archive (SRA) under accession number PRJNA626576. Metagenome data are available from the NCBI SRA under SRR12444714, SRR12444882, and SRR12445281. The OTUs representative sequences are deposited in NCBI GenBank under accession numbers MT409044-MT409096.

## Results

### Basic Information on Primers

Two widely used primer sets, Ntsp-amoA 162F/359R and comaA/B-244f/659r, were selected in this study. Ntsp-amoA 162F/359R contains high degeneracy (5 out of 24 base pairs) and is the first primer pair designed to simultaneously target both clade A and B comammox *Nitrospira*, generating a 198 bp-length fragment between the position of 162–182 and of 339–359 in comammox *amoA* gene (Figure 1 and Table 1). It has been widely used in comammox community analysis from various environments, such as freshwater biofilms, brackish lake sediments, rice rhizospheres, rice paddy soils, forest soils, wastewater treatment plants, and drinking water treatment plants (Wang Y. et al., 2017; Fowler et al., 2018).

The comaA/B-244f/659r primer mixtures consist of separately synthesized versions of the six respective sets of forward and reverse primers, with each primer containing only one or no base ambiguity (Table 1). They were designed to differentiate between (comaA-244f/659r for) clades A and (comaB-244f/659r for) clade B comammox *amoA* sequences within the *Nitrospira* genus (Table 1), amplifying a comammox *amoA* gene amplicons with a size of 415 bp at binding regions between positions 244–261 and 643–659 (Figure 1). This primer set has been used for the detection of comammox *amoA* genes in nitrification reactors (Roots et al., 2019), agricultural soils (Li et al., 2019; Liu et al., 2019; Wang et al., 2019), wetland (Shah and Wang, 2019), forest soil (Shi et al., 2018) and wastewater treatment plant (Spasov et al., 2019).

### PCR Results of Comammox *amoA* Genes

In order to compare the efficiency of two primer sets in amplifying comammox *amoA* genes and their ability to reveal the diversity of comammox community, PCR and agarose gel electrophoresis were conducted with the two primer sets for three typical wetland soils from the Tibetan Plateau and the Northeast Plain of China.

**FIGURE 2:**
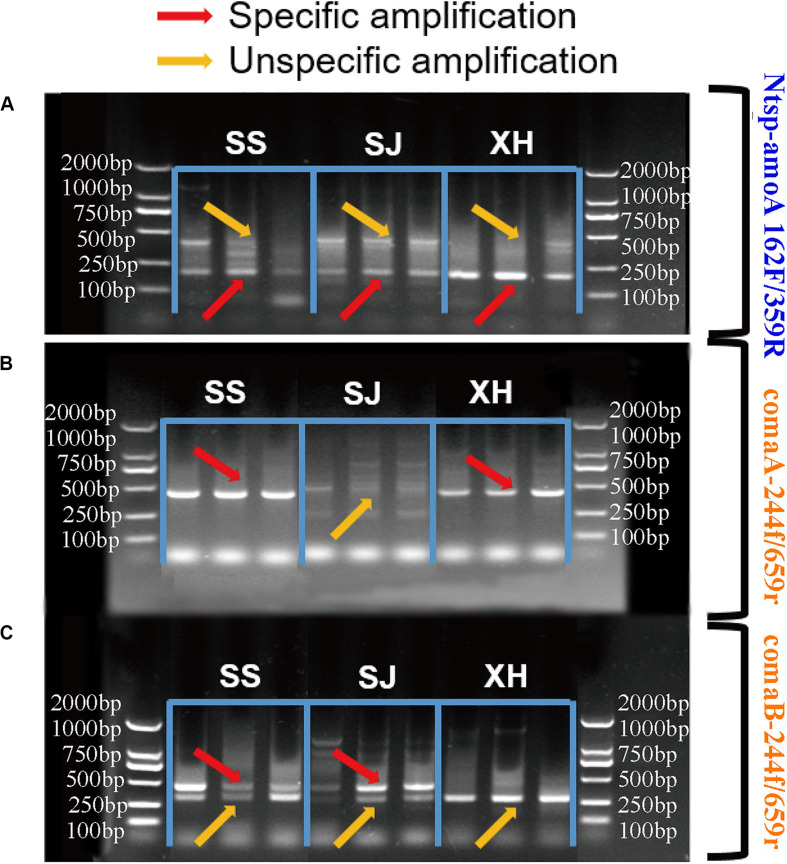
Agarose gel electrophoresis of PCR amplification products **(A)** with primer sets Ntsp-amoA 162F/359R, **(B)** comaA-244f/659r and **(C)** comaB-244f/659r in SS (Sangsang), SJ (Sanjiang) and XH (Xianghai) wetland soil samples. The red and yellow arrows represented specific amplification and non-specific amplification, respectively.

In the case of the primer set Ntsp-amoA 162F/359R, target PCR products with 198 bp length and non-specific amplification with around 500 bp length were both detected in all samples (Figure 2A). Increasing the annealing temperature could not eliminate this non-specific amplification (data not shown). This finding was similar to previous studies, showing that multiple products with correct and unexpected product sizes were both found in the agarose gel of qPCR products obtained with Ntsp-amoA 162F/359R (Beach and Noguera, 2019).

Regarding to the primer set comaA/B-244f/659r, comammox clade A *amoA* genes were detected in SS and XH wetland soils with high specificity (Figure 2B). The dim fragment with around 415 bp-long were observed in the SJ samples (Figure 2B), but no comammox *amoA* genes were identified by high-throughput sequencing from this dim fragment (Supplementary Table S3). In the clade B assay, the expected fragments were successfully amplified from SS and SJ samples with primer comaB-244f/659r (Figure 2C), but non-specific products of various sizes were also detected in the same samples (Supplementary Table S3), which was consistent with previous studies (Beach and Noguera, 2019). Primer comaB-244f/659r failed to amplify comammox clade B *amoA* genes in XH samples, while it produced a clear non-specific band approximately 350 bp in length, which was challenging to distinguish because it was very close to the expected 415 bp target size (Figure 2C). Numerous factors such as choice of annealing temperature, cycle times, DNA concentration and template preference might affect PCR efficiency (D’Amore et al., 2016; Gohl et al., 2016; Nilsson et al., 2018). However, after considerable efforts in adjusting the annealing temperature (varied in small increments from 48 to 60°C), concentration of template DNA (40, 20, and 1 ng DNA/reaction) and cycle times (from 30 to 35) of the PCR procedure, we still failed to get a clear target PCR band for the clade A in SJ and clade B in XH, and the non-specific products in our PCR results still could not be eliminated.

### Diversity of Comammox *amoA* Genes

The comammox *amoA* gene PCR products amplified with the primer pairs of Ntsp-amoA 162F/359R and comaA/B-244f/659r were sequenced on the Illumina MiSeq and HiSeq platforms, respectively. After quality control, we obtained a total of 2,669,629 comammox *amoA* gene sequences (a mixture of clades A and B) with Ntsp-amoA 162F/359R, and 776,017 gene sequences of clade A and 802,915 gene sequences of clade B with comaA-244f/659r and comaB-244f/659r, respectively. Rarefaction curves showed that the sequencing depth was sufficient for diversity analysis of comammox *amoA* genes (Supplementary Figure S1). Each data set was then trimmed to 16,109 reads per sample for the following community composition analysis. In total, 1,115 OTUs at a cutoff of 95% sequence similarity were obtained for all comammox *amoA* genes with the primer pair of Ntsp-amoA 162F/359R for the three wetland soils, while 70 and 105 OTUs were detected for the clade A and clade B comammox with the primer pairs of comaA-244f/659r and comaB-244f/659r, respectively.

**FIGURE 3:**
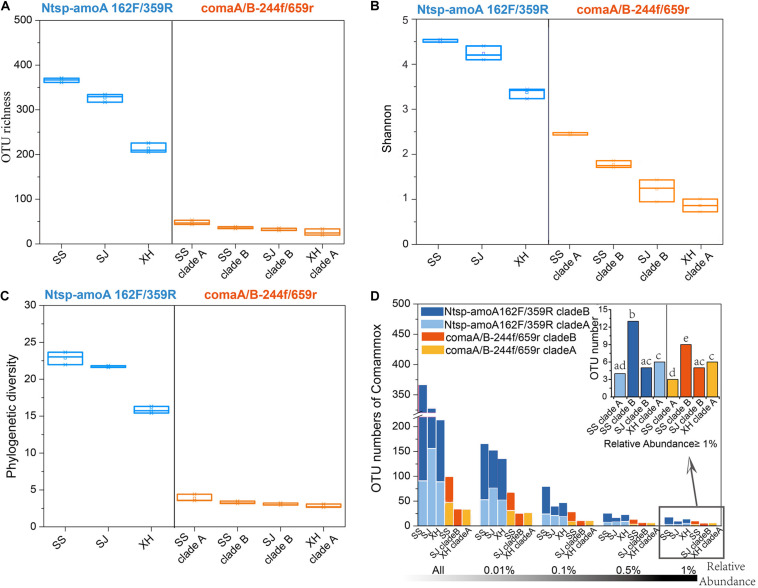
**(A)** OTU richness, **(B)** Shannon index, and **(C)** phylogenetic diversity of comammox communities in three wetland soils using primer Ntsp-amoA 162F/359R (blue) and comaA/B-244f/659r (orange). **(D)** OTU numbers of comammox *amoA* gene sequences amplified by primer Ntsp-amoA 162F/359R (blue) and comaA/B-244f/659r (orange) with RA greater than 0.01, 0.1, 0.5, and 1% in the total reads of each sample. A bar graph on the top right indicated the difference between OTU numbers of the dominant OTUs with RA ≥ 1.0% amplified by two primer sets. Bars do not share the same letter considered to be different (*P* < 0.05).

With primer set of Ntsp-amoA 162F/359R, the diversity indices (OTU numbers, Shannon index, phylogenetic diversity) indicated that the comammox community with the highest diversity was found in the SS site, followed by the SJ and XH sites (Figures 3A–C and Supplementary Table S1). Similar results were found with the primer set comaA/B-244f/659r (SSB > SSA > SJB > XHA) (Figures 3A–C and Supplementary Table S1), but as mentioned above, clade A and clade B failed to be amplified using comaA/B-244f/659r in SJ and XH, respectively (Figures 2B,C). However, compared to comaA/B-244f/659r, Ntsp-amoA 162F/359R tended to identify more ASVs (Supplementary Table S5) or detect a greater richness of OTUs for the common clades that both primer sets could successfully amplify (i.e., clades A and B in SS, clade B in SJ and clade A in XH) from the three wetland soils. To further test this pattern, we selected the OTUs with RA greater than 0.01, 0.1, 0.5, and 1% from the total reads of each sample, and then compared the coverage of dominant and rare OTUs of comammox *amoA* by using these two primer sets. Results showed that for the clades that can be captured by two primer sets, Ntsp-amoA 162F/359R had significantly higher (*P* < 0.05) number of OTUs (RA ≤ 0.5%) than comaA/B-244f/659r (Figure 3D). However, for the dominant OTUs with RA ≥ 1.0%, the two primer sets identified similar OTU numbers (*P* > 0.05) for the clade A from SS and XH and for the clade B from SJ (Figure 3D). This indicated that the two primer sets normally detected a similar number of dominant OTUs with RA ≥ 1.0%, and Ntsp-amoA 162F/359R tended to cover more rare OTUs with 0.1% ≤ RA < 0.5% than comaA/B-244f/659r.

### Comammox Community Composition and Phylogeny

**FIGURE 4:**
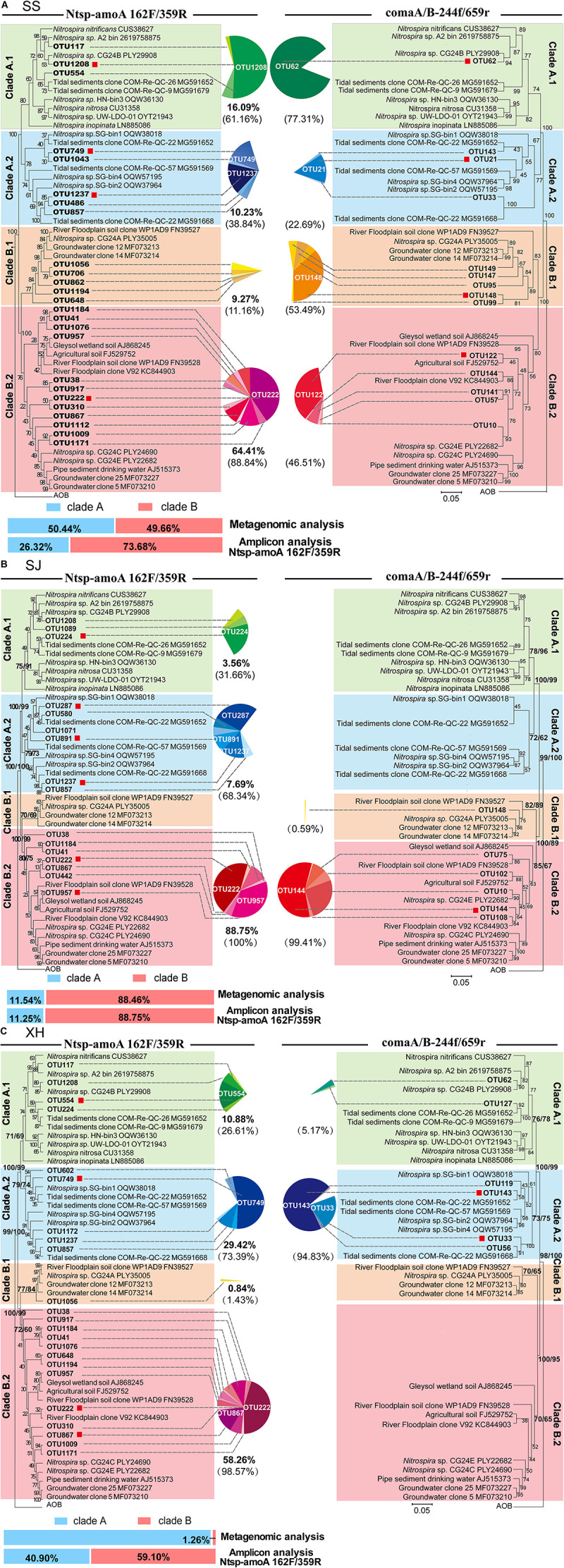
Maximum-likelihood phylogenetic trees based on representative sequences of the OTUs (RA > 0.5% of each sample) and 77 reference sequences using Ntsp-amoA 162F/359R (left) and comaA/B-244f/659r (right) from panel **(A)** SS, **(B)** SJ, and **(C)** XH samples. A Sequence from betaproteobacterial *amoA* was used as a outgroup. Bootstrap values in the maximum-likelihood and phylogenetic neighbor-joining trees calculated based on 1,000 trials were separated by a slash. Sequences obtained in this study were marked in bold. Red squares indicate the OTUs that its RA was larger than 0.5% of each clade. Sequences obtained from different clades are indicated by color symbols for clade A.1 (green), clade A.2 (blue), clade B.1 (orange) and clade B.2 (pink). Pie charts illustrate the proportion of OTUs in each clade with the bold data under the pie chart presenting the account of each group in the total sequences that RA was larger than 0.5%. The data in parentheses indicate the proportion of each group in clade A or B. Metagenomics and amplicon (Ntsp-amoA 162F/359R) results of clade A (blue) and B (red) proportion in each sample are in the lower-left corner.

Phylogenetic trees of comammox *amoA* OTUs (RA > 0.5% of each sample) from three wetland samples obtained with the two primer sets were constructed separately using the maximum-likelihood method (Figure 4). Clearly, separation of the known clades A and B, with each clade being further divided into two monophyletic groups, sub-clades A.1, A.2 and sub-clades B.1, B.2, respectively, was observed in the phylogenetic trees (Figure 4). The division of clade B into two monophyletic groups was supported by high bootstrap values (ranging from 70 to 85%) and was also confirmed with neighbor-joining phylogenetic trees.

Both clades A and B of comammox were detected in the SS samples with the two primer sets (Figure 4A). Results of Ntsp-amoA 162F/359R showed that clade B predominated at SS with 73.68% of the total comammox *amoA* gene sequences, within which 87.4% belonged to clade B.2. The proportion of clade A.1 was slightly higher than that of clade A.2, and there was one dominant OTU (OTU1208) in clade A.1 accounting for 52.1% of total clade A comammox *amoA* gene sequences in SS. Similarly, with the primer set of comaA/B-244f/659r, it showed higher proportion of clade A.1 than clade A.2 within clade A and a highly predominant OTU (OTU62) in clade A.1 (Figure 4A). The high sequence similarity (96.5%) between OTU1208 detected by Ntsp-amoA 162F/359R and OTU62 detected by comaA/B-244f/659r suggested they could most likely clustered into the same OTU (Supplementary Table S2). But unlike the results of former primer pair, the latter revealed a slightly higher proportion of clade B.1 than that of clade B.2 in the SS wetland.

As mentioned above the primer set of comaA/B-244f/659r failed to amplify clade A in SJ and clade B in XH. In contrast, both clades could be successfully amplified using the primer set of Ntsp-amoA 162F/359R (Figures 2B,C, 4B,C). However, the two primer sets demonstrated relatively similar structure patterns for the common clades (clade B in SJ and clade A in XH) that successfully amplified with both of the two primer sets (Figure 4B,C). For instance, both two primer sets revealed that, in SJ, > 99% comammox *amoA* gene sequences of the clade B belonged to clade B.2 that included the two most abundant OTUs (Figure 4B). Similarly, in XH, clade A.2 was revealed as the dominant lineage for the clade A comammox *amoA* genes using both of the two primer sets (Figure 4C). The most abundant OTU detected with Ntsp-amoA 162F/359R (OUT 749) and comaA/B-244f/659r (OUT 143) shared high sequence similarity (97.2%) in the overlapped areas (Supplementary Table S2), indicating the general consistency of two primer sets in identifying the most abundant OTU (Figure 4C).

### Metagenomic Analysis

Due to the low relative abundance of comammox in the soil microbial community in each sample, only 2–5 comammox *amoA* gene fragments with the length ≥338 bp were retrieved from the metagenomic data, which could not represent the diversity of comammox in each soil sample as shown above (Figure 4). Therefore, a hybrid annotation strategy was used for the short-read screening (Liu et al., 2020) to determine the relative abundances of clade A and clade B in each soil sample, and found that the relative abundance of clade A and clade B is 50.44% (74 reads) and 49.66% (73 reads) in SS (Figure 4A and Supplementary Table S4), 11.54% (3 reads) and 88.46% (23 reads) in SJ (Figure 4B and Supplementary Table S4), and 98.74% (314 reads) and 1.26% (4 reads) in XH (Figure 4C and Supplementary Table S4), respectively.

### Coverage of Two Primer Sets *in silico* Analysis

As mentioned above, comaA/B-244f/659r still failed to capture comammox *amoA* clade A in SJ samples and clade B in XH samples even after our great efforts in changing annealing temperature, concentration of template DNA and cycle times. Thus, we explored whether the mismatch of primer-template was the reason for the inability of comaA/B-244f/659r to amplify clade A in SJ and clade B in XH (Figures 2B,C) that, however, could be successfully amplified by the primer pair of Ntsp-amoA 162F/359R. Because there is an overlap between the PCR products obtained by the two primer sets (Figure 1), i.e., the 198 bp-long fragment generated by Ntsp-amoA 162F/359R contains the region of 244–261 bp of comammox *amoA* gene, which is exactly the targeted position designed for the forward primer of comaA/B-244f/659r (Figure 1). Thus, it allows us to analyze the sequence diversity of the region of 244–261 bp within 198 bp-long gene fragments amplified by Ntsp-amoA 162F/359R, comparing with the *in silico* coverage of forward primer of comaA/B-244f/659r.

**FIGURE 5:**
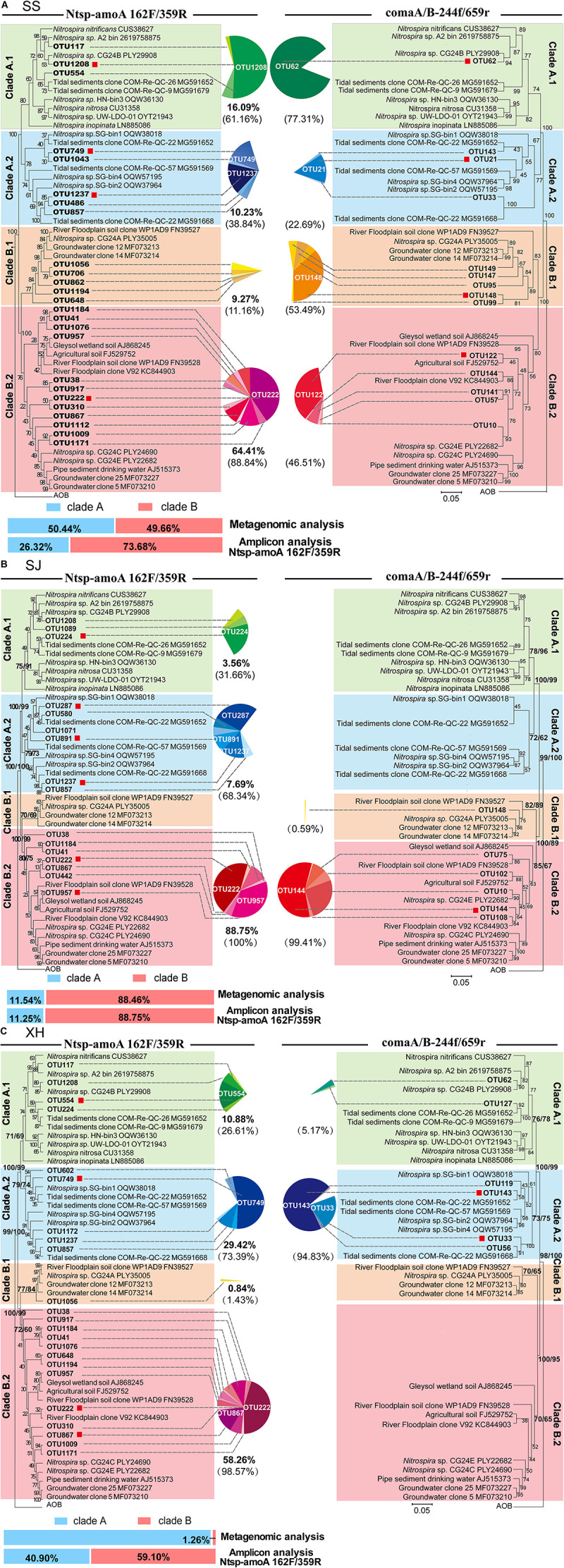
Primer-target mismatch analysis for former primer comaA/B-244f and the missed comammox *amoA* sequences (clade A in SJ and clade B in XH samples) amplified by Ntsp-amoA 162F/359R (RA > 0.5%). 0 and 1 mismatch indicate former primer comaA/B-244f binding to corresponding region in comammox *amoA* gene sequences amplified by Ntsp-amoA 162F/359R without base mismatch and with 1 base mismatch, respectively.

It is odd that up to 72.03% of the 198 bp length fragments of clade A *amoA* gene produced by Ntsp-amoA 162F/359R in the SJ wetland harbor a region of 244–261 bp, sharing 100% sequence identity with the forward primer of comaA-244f/659r (Figure 5). Similarly, 86.41% of the 198 bp length gene fragments of clade B *amoA* gene amplified by Ntsp-amoA 162F/359R in XH contain a region of 244–261 bp, sharing 100% sequence identity with the forward primer of comaB-244f/659r (Figure 5). Allowing one mismatches, the forward primers comaA/B-244f could cover 100% of sequences produced by Ntsp-amoA 162F/359R. However, as mentioned above, comaA/B-244f/659r could not successfully amplify the clade A and clade B comammox *amoA* genes in the SJ and XH wetland, respectively (Figures 2B,C, 4C). The above results clearly suggest that partial amplification of comaA/B-244f/659r was not sorely attributed to the low *in silico* coverage of its forward primer mixture. Additionally, due to the limited length of PCR products of Ntsp-amoA 162F/359R, there is no information on sequence match between reverse primer of comaA/B-244f/659r and the position of 643–659 bp within the clade A/B *amoA* gene lineage detected by Ntsp-amoA 162F/359R.

## Discussion

A critical step in understanding the ecological characteristics and functional importance of comammox communities is choosing suitable primers that have good coverage, high specificity, and sufficient amplification efficiency without bias. In order to provide reliable evidence for primer selection, we compared the performance of two of the most commonly used primer sets Ntsp-amoA 162F/359R and comaA/B -244f/659r for the detection of comammox communities in three typical wetland soils. Despite limited samples, we still found that two primer sets revealed a broadly similar diversity and community distribution of dominant OTUs, but there are also differences in terms of identifying the clades with relative low abundance and rare OTUs, as well as generating non-specific amplification. We then explored the possible reasons underlying the observed differences.

For the common clades that could successfully be amplified by both sets of primers (i.e., clade A and B in SS, clade B in SJ, and clade A in XH) from the three wetland soils, it was not surprising that the number of dominant OTUs obtained by Ntsp-amoA 162F/359R and comaA/B-244f/659r was similar (Figure 3D). However, larger OTU numbers, hence greater diversity indices were obtained when detecting with Ntsp-amoA 162F/359R compared to comaA/B-244f/659r (Figures 4A–C). Since all the *amoA* gene reads were strictly trimmed and quality-controlled before OTU clustering, and all of rare OTUs (0.1% ≤ RA < 0.5%) captured by Ntsp-amoA 162F/359R or by comaA/B-244f/659r contained more than 48 sequences, it appears that these rare OTUs were real biological sequences and not produced by sequencing errors. Furthermore, this conclusion was further supported by the analysis based on ASVs (Nearing et al., 2018; Forster et al., 2019), which also demonstrated much more ASVs were obtained by Ntsp-amoA 162F/359R than by comaA/B-244f/659r (Supplementary Table S5).

**FIGURE 6:**
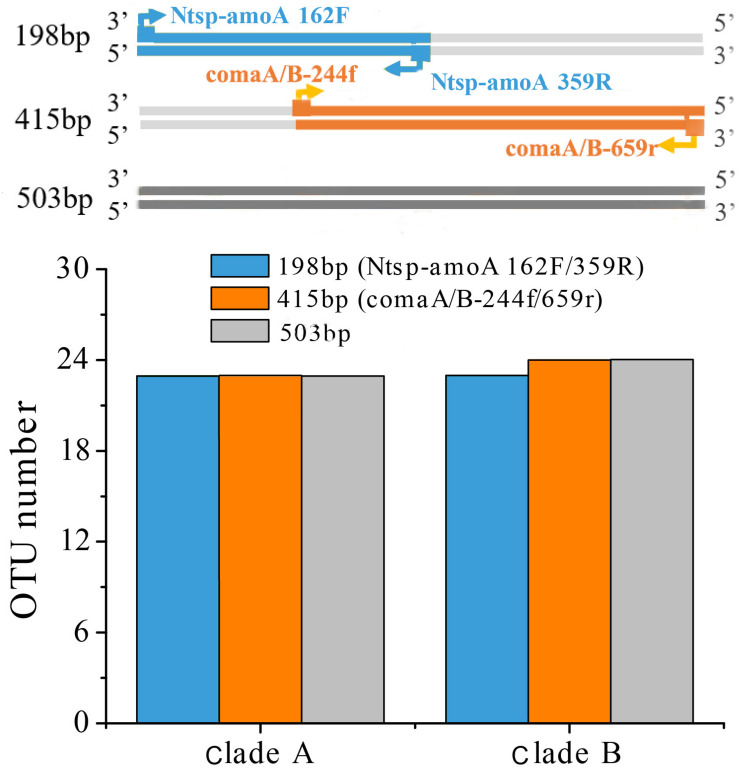
OTU cluster analysis with 95% similarity cutoffs of comammox *amoA* gene sequences for clade A (115 sequences) and B (112 sequences) with length ≥503 bp from the NCBI-nr database covering both regions amplified by two primer sets at 198 bp-length (Ntsp-amoA 162F/359R), 415 bp-length (comaA/B-244f/659r) amplification regions and 503 bp-length regions.

A reasonable supposition is that the different sequence variance of regions amplified by the two primer sets may have affected the OTU clustering and subsequent diversity analysis. Even if the sequences were the same, OTU numbers obtained in different amplified regions might be different due to their different average genetic distance. Therefore, we searched all the comammox *amoA* genes with length ≥503 bp from the NCBI-nr database covering both regions amplified by two primer sets to analyze the diversity of 198 bp length, 415 bp length amplification regions and 503 bp length regions. A total collection of 115 sequences of clade A and 112 sequences of clade B was then clustered into OTUs with 95% sequence similarity for the OTUs comparison analysis. We found that the number of OTUs (both clades A and B) among the two amplicon regions and the whole gene length showed little differences, indicating that it was not the differently amplified regions of the two primer sets resulted in higher diversity of the *amoA* gene sequences captured by Ntsp-amoA 162F/359R (Figure 6). Although only 237 sequences were insufficient to represent the global diversity of comammox *amoA* genes, we regarded this method as a random sampling. We also recognize that more comammox *amoA* gene sequences are required to further confirm our findings.

For the failure of comaA/B-244f/659r to amplify some comammox *amoA* clades in SJ and XH, metagenomic results suggested that this may be due to the low-abundance of target genes in wetland soil samples. For example, clade A in SJ and clade B in XH that were not detected with comaA/B-244f/659r, accounted for only 11.54 and 1.26% of the total comammox *amoA* genes in each sample, respectively, according to metagenomic results (Figures 4B,C), indicating that the unsuccessful amplification might be attributed to the low abundance of target clades. By contrast, Ntsp-amoA 162F/359R could detect these low-abundance clades, suggesting its potential advantages of identifying rare OTUs. Wang et al. (2019) reported a similar phenomenon that primer sets comaA/B -244f/659r failed to amplify comammox *amoA* for most DNA samples from fertilized soil where the abundance of comammox *amoA* genes ranged from 1.3 × 10^5^ to 5.5 × 10^5^ copies/g soil yielded with the primer set Ntsp-amoA 162F/359R (Wang et al., 2019). In addition, primer-target mismatch is likely another factor to prevent detection of clade A in SJ and clade B in XH. Silico analysis indicated that, compared to forward primer comaA/B-244f, 27.97% of clade A comammox *amoA* genes in SJ and 13.59% of clade B in XH captured by Ntsp-amoA 162F/359R contained one mismatch (Figure 5). A single mismatch in a primer sequence can affect the efficiency of PCR amplification and even lead to an underestimation of the gene copy number by 1,000 times (Bru et al., 2008). But given that majority of missed sequences shared 100% exact match with forward primer comaA/B-244f, primer-target mismatch might not the main cause. Moreover, the impact of reverse primers could not be ruled out as we were not able to analyze the silico binding region of reverse primers. But due to the limitation in sample type and numbers, this conclusion warrants further investigation.

It is worth noting that although the RA of each clade obtained with Ntsp-amoA 162F/359R was consistent with that retrieved from metagenome data in SJ samples (Figure 4B), Ntsp-amoA 162F/359R vastly overestimated the abundance of comammox clade B in XH samples. Comparison of metagenomic and amplicon data showed a dramatic increase in the RA of comammox clade B from 1.26% (metagenomic results) to 59.70% of comammox *amoA* genes in XH samples (Figure 4C). The overestimation of comammox clade B also appeared in SS samples, where clade B accounted for 73.68% with primer set Ntsp-amoA 162F/359R, whereas actually 49.66% of comammox *amoA* genes was detected as clade B using PCR-free metagenomic analysis (Figure 4A). Therefore, the results of relative abundance for clades A and B comammox *amoA* genes obtained by Ntsp-amoA 162F/359R should be regarded with caution unless verified by metagenomic analysis of the same samples.

Despite that comammox *amoA* genes can be successfully amplified in some samples using comaA/B -244f/659r and Ntsp-amoA 162F/359R, these two primers yielded non-specific amplification in all our tests, which could not be eliminated by adjusting annealing temperature, concentration of template DNA and cycle numbers of PCR procedure. This observation was consistent with previous studies (Pjevac et al., 2017; Beach and Noguera, 2019). The ubiquitous non-specific amplification suggested that we should be careful to avoid overestimation of comammox *amoA* genes when performing qPCR with the two primer sets. One possible reason for non-specific amplification was the high similarity between target fragments and some other naturally occurring gene fragments in the microbial community, and that both of them can match to the primer binding sites (Supplementary Table S3). Another possible reason would be the presence of ambiguous bases in the primer sets that could impair PCR specificity (Linhart and Shamir, 2002). For example, only one small non-target PCR band was observed (Figure 2B) in three wetland soil samples with primer sets comaA -244f/659r for clade A (without any ambiguous bases). While, several non-target PCR bands were observed with primer sets comaB -244f/659r (with one ambiguous base) for clade B and Ntsp-amoA 162F/359R (with five ambiguous bases) (Figures 2A,C).

Taken together, our study clearly demonstrated that the two widely used primer sets of comammox *amoA* genes performed equally in terms of revealing the composition of the clades with relative high abundance and showing similar distribution of the dominant OTUs in the wetland soil samples. The primer pair comaA/B-244f/659r has prominent advantage in the aspect of studying the separate clade of comammox *amoA* genes and in generating fewer non-target gene sequences. In comparison, Ntsp-amoA 162F/359R has better performance for capturing comammox clades with low RA and for revealing more information about rare OTUs in community diversity. However, it is also more prone to forming non-specific PCR-band and non-target gene sequences via Illumina sequencing, and often greatly overestimating the clades with low RA. Due to the generally very low abundance of comammox in soil microbial community, the metagenome sequencing as deep as 40 Gbp could only assemble a few long comammox *amoA* gene fragments from each sample, screening of the short reads proved to be a reliable alternative strategy for revealing the true community structures of comammox in each sample. All considered, we urge caution in interpreting results based on these primers and suggest the development of more efficient and specific primer sets, as well as the necessary verification work. Future studies may also consider the potential benefits of using PCR-free deep metagenomic sequencing to investigate highly diverse comammox communities in natural environments.

## Data Availability Statement

The datasets presented in this study can be found in online repositories. The names of the repository/repositories and accession number(s) can be found below: https://www.ncbi.nlm.nih.gov/genbank/, MT409044–MT409096; https://www.ncbi.nlm.nih.gov/, SRR12444714, SRR12444882, and SRR12445281.

## Author Contributions

CL wrote the manuscript. WQ, HX, and BJ revised the manuscript. SX analyzed experimental results. XT carried out the experiments. LK and XW analyzed sequencing data. JC assisted with Illumina sequencing. JS and JA revised the manuscript. HQ designed the experiments. BW substantial contributed to the conception and design. All authors contributed to the article and approved the submitted version.

## Conflict of Interest

The authors declare that the research was conducted in the absence of any commercial or financial relationships that could be construed as a potential conflict of interest.

## Footnotes

1 http://www.bioinformatics.babraham.ac.uk/projects/fastqc/

2 http://qiime.sourceforge.net

3 http://fungene.cme.msu.edu/

4 http://iqtree.cibiv.univie.ac.at/

5 https://github.com/najoshi/sickle
