# Supplementary material for: Evaluation of Two Primer Sets for Amplification of Comammox Nitrospira amoA Genes in Wetland Soils
Source: Front Microbiol. 2020 Sep 30;11:560942. doi: 10.3389/fmicb.2020.560942 (PMC7555835; doi:10.3389/fmicb.2020.560942)
Supplement: Supplementary file 2 [file Table_2.docx]

Supporting Information

**Evaluation of Two Primer Sets for Amplification of Comammox *Nitrospira amoA* Genes in wetland soils**

**Chenshuo Lin^1, 2^, Hang Xu^2^, Wei Qin^3^, Shaoyi Xu^4^, Xiufeng Tang^5^, Lu Kuang^5^, Xinxin Wang^5^, Bin Jiang^6^, Junhui Chen^1^, Jun Shan^5^, Jonathan Adams^7^, Hua Qin^1,^ *, Baozhan Wang^5, 8,^ ***

^1^Key State Laboratory of Subtropical Silviculture, Zhejiang A & F University, Lin'an, Hangzhou 311300, China

^2^Key Laboratory of Integrated Regulation and Resource Development on Shallow Lake of Ministry of Education, College of Environment, Hohai University, Nanjing 210098, China

^3^Department of Microbiology and Plant Biology, University of Oklahoma, Norman, OK, United States

^4^Key Laboratory of Environment Remediation and Ecological Health, Ministry of Education, College of Environmental Resource Sciences, Zhejiang University, Hangzhou, 310058, China

^5^State Key Laboratory of Soil and Sustainable Agriculture, Institute of Soil Science, Chinese Academy of Sciences, Nanjing, Jiangsu Province, 210008, China

^6^Agricultural Genomics Institute at Shenzhen, Chinese Academy of Agricultural Sciences, Shenzhen, 518120, China.

^7^School of Geography and Ocean Science, Nanjing University, Nanjing 210023, China.

^8^Key Lab of Microbiology for Agricultural Environment, Ministry of Agriculture, College of Life Sciences, Nanjing Agricultural University, Nanjing, 210095, China.

***Correspondence:**

Dr. Prof. Baozhan Wang

bzwang@njau.edu.cn

Dr. Prof. Hua Qin

qinhua@zafu.edu.cn.

**Keywords: Comammox, *amoA*, Ntsp-amoA 162F/359R, comaA/B-244f/659r, PCR.**


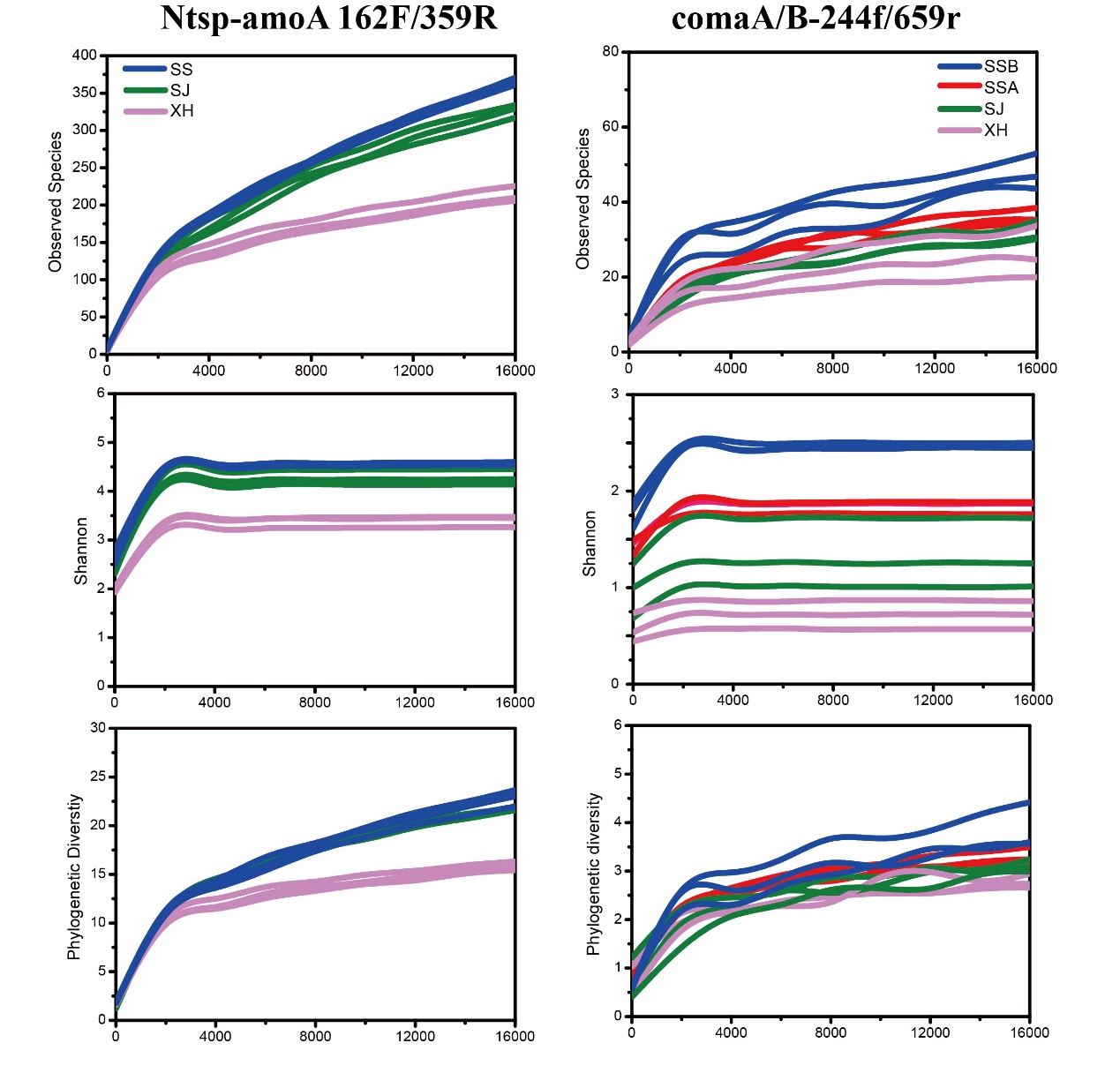


**Figure S1**. Rarefaction curves of individual richness index for wetland samples, which were amplified by primer comaA/B-244f/659r and primer Ntsp-amoA 162F/359R, respectively.

**Table S1.** OTU numbers of comammox *amoA* amplified by primer Ntsp-amoA 162F/359R (blue) and comaA/B-244f/659r (orange) with RA greater than 0.01 %, 0.1 %, 0.5 %, and 1 % in the total reads of each sample. The proportion of each clade in corresponding total sequence was calculated by dividing within brackets. (Excel file format)

**Table S2.** Sequences similarity analysis for the representative sequences of OTUs (RA＞0.5%) amplified by primers comaA/B-244f/659r and Ntsp-amoA 162F/359R in their overlapped areas. Only the results with a similarity higher than 95% were shown in the table. (Excel file format)

**Table S3.** Ten sequences of non-specific bands which was very close to the expected 415 bp target size and were covered by comaA/B-244f/659r in the SS samples. (Excel file format)

**Table S4.** The proportion of Comammox *amoA* clade A and B in SJ, SS and XH samples from metagenomic and amplicon (Ntsp-amoA 162F/359R) results.

**Table S5.** The average number of ASVs captured by Ntsp-amoA 162F/359R and comaA/B-244f/659r in SJ, SS and XH wetland soil samples.
